# Supplementary material for: Complex‐centric proteome profiling by SEC‐SWATH‐MS
Source: Mol Syst Biol. 2019 Jan 14;15(1):e8438. doi: 10.15252/msb.20188438 (PMC6346213; doi:10.15252/msb.20188438)
Supplement: Supplementary file 8 — Dataset EV7 [file MSB-15-e8438-s008.zip › feature_plots_string/Q92973.pdf]

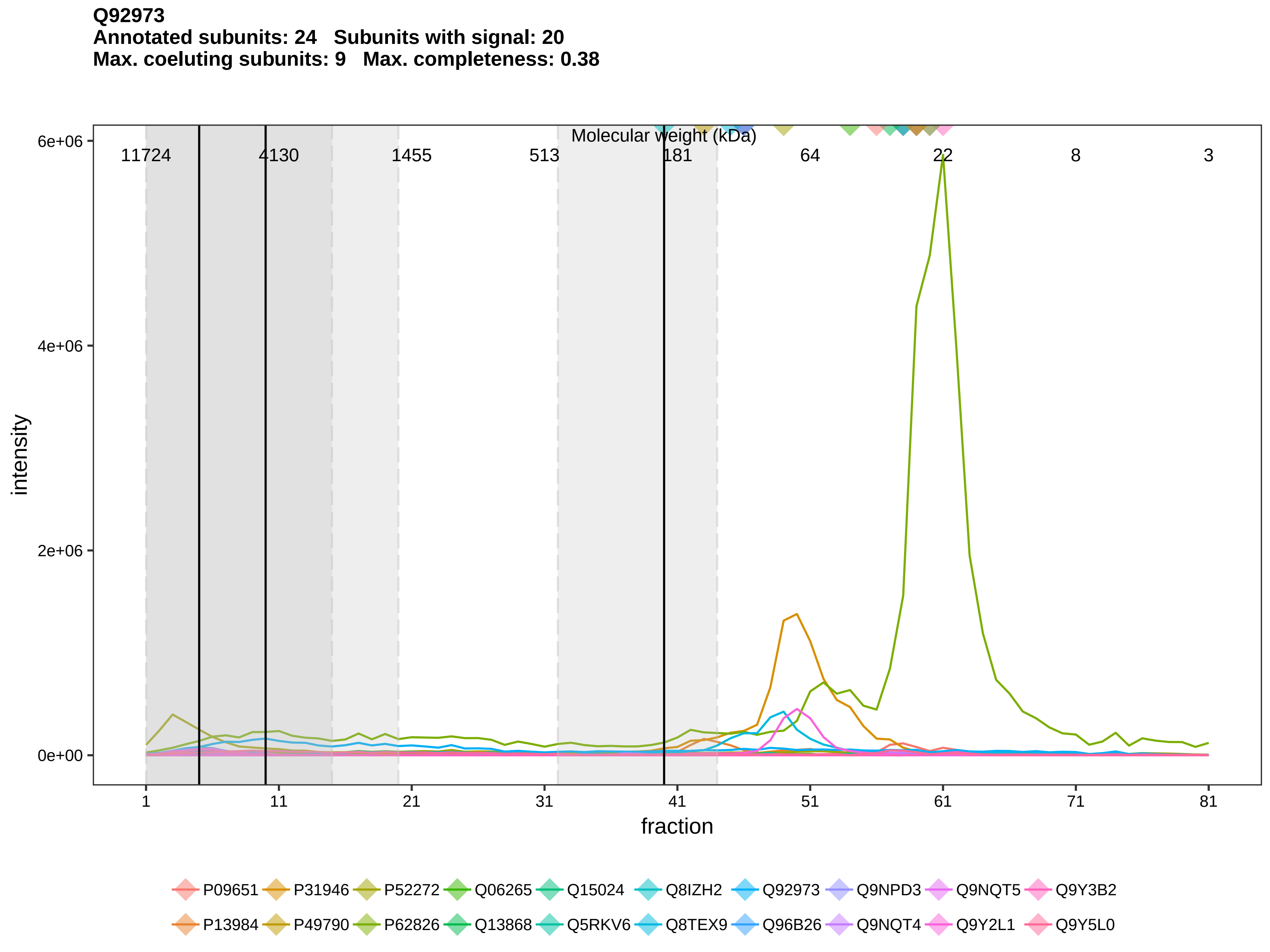
Q92973
Annotated subunits: 24 Subunits with signal: 20
Max. coeluting subunits: 9 Max. completeness: 0.38
Molecular weight (kDa)
11724
4130
1455
513
181
64
22
8
3
6e+06
4e+06
2e+06
0e+00
intensity
1
11
21
31
41
51
61
71
81
fraction
P09651
P13984
P31946
P49790
P52272
P62826
Q06265
Q13868
Q15024
Q5RKV6
Q8IZH2
Q8TEX9
Q92973
Q96B26
Q9NPD3
Q9NQT4
Q9NQT5
Q9Y2L1
Q9Y3B2
Q9Y5L0
